# Fluorescence Enhancement Using Bimetal Surface Plasmon-Coupled Emission from 5-Carboxyfluorescein (FAM)

**DOI:** 10.3390/mi9090460

**Published:** 2018-09-12

**Authors:** Nhu Hoa Thi Tran, Kieu The Loan Trinh, Jun-Ho Lee, Won Jung Yoon, Heongkyu Ju

**Affiliations:** 1 Department of Nano-Physics, Gachon University, 1342 Seongnam-daero, Sujeong-gu, Seongnam-si, Gyeonggi-do 13120, Korea; ttnhoa@hcmus.edu.vn; 2 Gachon Bionano Research Institute, Gachon University, 1342 Seongnam-daero, Sujeong-gu, Seongnam-si, Gyeonggi-do 13120, Korea; 3 Department of BioNano Technology, Gachon University, 1342 Seongnam-daero, Sujeong-gu, Seongnam-si, Gyeonggi-do 13120, Korea; tktloan@gmail.com; 4 Laser & Opto-electronics Team, Korea Electronics Technology Institute, Seongnam-si, Gyeonggi-do 13509, Korea; junholee@keti.re.kr; 5 Department of Chemical and Bio Engineering, Gachon University, 1342 Seongnam-daero, Sujeong-gu, Seongnam-si, Gyeonggi-do 13120, Korea; wjyoon@gachon.ac.kr; 6 Neuroscience Institute, Gil Hospital, Incheon 405-760, Korea

**Keywords:** fluorescence enhancement, surface plasmon resonance, 5-carboxyfluorescein, nano-biophotonics, optical biosensors

## Abstract

We demonstrate the enhancement of fluorescence emission from a dye, 5-carboxyfluorescein (FAM), which couples with surface plasmons at the spectral channels of excitation and emission. Experiments and calculations revealed that bimetallic (gold-silver) plasmon, as compared to the monometallic ones, allowed such coupling to be enhanced, at both the spectral channels. We achieved the maximum fluorescence enhancement level of 46.5-fold, with markedly high reproducibility (coefficient of variation ~ 0.5%) at a FAM concentration of 10 nM. We also found that higher fluorescence enhancement was more likely to be reproducible. This encourages the use of this technology for practical applications in fluorescence-based biochemical assays. Moreover, we investigated a FAM concentration-dependent enhancement of fluorescence. It was found that fluorescence enhancement decreased and saturated at above 10 nM concentration possibly due to partial photo-bleaching of FAM molecules.

## 1. Introduction

Fluorescence is one of the widely used technologies for qualitative and quantitative detection of biomolecules in the biochemical sensing and life science industry. In practical applications of fluorescence techniques, there has been a continual demand for an increase in the signal-to-noise ratio (SNR) in the detection of biomolecules of low concentrations [1,2,3,4]. To enable a high SNR in fluorescence detection, a variety of nanostructures have been introduced to enhance fluorescence, such as plasmonic ones [5,6,7,8,9,10,11,12], photonic crystal systems [13] and alumina nanopores [14] for biomolecular assays. A fluorescence intensity enhancement has been obtained with porous structures (7- to 10-fold) [13,15], with optical interference mirrors (about 40-fold) [16], with silver (Ag) nanoparticles (6- to 25-fold) [14,17,18,19], with Ag island films on metallic mirrors (50-fold) [20], in the plasmonic resonance enhanced fluorescence energy transfer (20-fold) [21], and by the prism-based surface plasmon resonance (SPR) (10- to 47-fold), similar to the Kretschman-Rather configuration for SPR excitation [22].

One efficient solution for SNR elevation is to use metallic structures brought in nanoscale proximity to dye molecules, for luminescence enhancement, which is referred to as the metal-enhanced fluorescence (MEF), in a broad sense [23,24,25,26,27,28,29,30,31]. Particularly, in SPR, the resonance state that results from the coherently phase-matched coupling between a collective oscillation mode of conduction electrons and a transverse magnetic (TM) mode of the electromagnetic wave (at a metal–dielectric interface), has been employed to effectively demonstrate fluorescence enhancement and photostability [32,33]. It is known that surface plasmons can couple with fluorescent dye molecules to enhance the dye excitation rate, at an excitation wavelength (λ_ex_) [34]. This is then followed by a near-field coupling of the excited state of dye molecules with the surface plasmons, at an emission wavelength of (λ_em_). This leads to directional radiation at λ_em_ into a transparent high index medium interfaced with the plasmonic metal film [5,6,7,8,9,10,23,24,35,36]. The directional radiation would favor fluorescence collection efficiency, which also contributes to the SNR elevation.

Recently, we have demonstrated the presence of highly reproducible and sensitive fluorescence assay of double-stranded DNAs, using the fluorescent dye of SYBR Green I, which was coupled with bimetal surface plasmons [36]. No photo-bleaching was observed but a very high reproducibility (coefficient of variation (CV) < 1%) was achieved, for a limit of detection (LOD) of 400 fg/μL DNA, which is still the lowest ever, to date.

In this study, we present the plasmonic enhancement of fluorescence from 5-carboxyfluorescein (FAM), which is one of the most widely used fluorescent dye with a high quantum yield, to label oligonucleotides. As in Reference [36], we used two structures of optical setups which were distinguishable by a dye excitation method, but they both permitted surface plasmon-coupled emissions to occur. Calculation of results revealed that bimetal surface plasmon (2 nm Au/50 nm Ag coating) provided a plasmonic field strength higher than monometallic cases, at the excitation and emission spectral channels of FAM. This led us to achieve a fluorescence enhancement factor ($\eta_{E}$) of 46.5-fold at maximum, which was also highly reproducible, with a CV of 0.5%. Interestingly, we found that higher fluorescence enhancement was accompanied with a lower CV (higher reproducibility), in the continuous thin film-based plasmonic technologies. We examined FAM concentrations (C_FAM_)-dependent fluorescence enhancement and observed an $\eta_{E}$ of more than 10-fold, at above C_FAM_ = 5 nM. In contrast to Reference [36], it was found that $\eta_{E}$ does not increase monotonically, which is a feature characteristic of FAM. This may imply that a part of its dye molecules undergoes photo-bleaching while the others contribute to fluorescence enhancement through a plasmonic-coupling.

## 2. Experimental Apparatus and Techniques

### 2.1. Experimental Setup for Fluorescence

**Figure 1:**
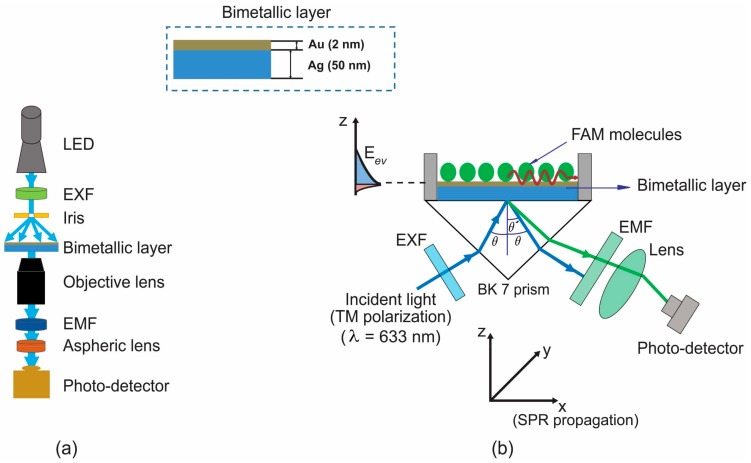
(**a**) A transmission geometry-based setup (T-mode) and (**b**) a prism-based set up (R-mode), for fluorescence enhancement detection. LED denotes a light emitting diode, EXF denotes an excitation filter, and EMF denotes an emission filter.

We utilized two optical setups with a transmission (T-mode) and a reflection (R-mode) geometries for fluorescence enhancement, similar to our previous work [36,37], as shown in Figure 1. Note that in this T-mode of the setup, which had the excitation source on one side of a plasmonic chip (opposite to a detector), the plasmon-coupled fluorescence emission was detected through the glass substrate. At the same time, the plasmonic metal film could also permit the transmission of excitation light through it. Thus, an emission filter was placed before a photodetector to ensure the detection of light at λ_em_. On the other hand, the R-mode setup was used to excite the SPR with the prism, whereby fluorescence, which is reflecting at an angle larger than the reflection angle of the excitation light, can be detected.

Polydimethylsiloxane (PDMS) prepolymer (Sylgard 184, Dow Corning, MI, USA) and a curing agent were purchased from Dow Corning, MI, USA. The ratio of the PDMS prepolymer to curing agent was 10:1 (*w/w*). The oven was heated up to 70 °C, for 1 h, to completely cure the PDMS. We bonded a PDMS mask with the prism base surface, for plasmonic metal coating. This mask also formed a part of the microchamber, which is supposed to contain a liquid buffer with dyes. The metallic thickness was optimized using numerical calculations, to maximize the electric field strength produced by the surface plasmons [36]. We simulated the case of monometallic and bimetallic layers of various thicknesses, i.e., monometallic layers of Au (30, 42, 52, and 60 nm thicknesses) and monometallic layers of Ag (30, 44, 52, and 60 nm thicknesses), as shown in Figure S1. This led us to choose the single and bimetallic layers of—a monometallic layer of Au (44 nm) and of Ag (52 nm), the bimetallic layers of Au (2 nm) and Ag (50 nm) as the best layers that produced the highest electric field enhancement. A thermal evaporation system (Daeki Hi-Tech Co. Ltd. Daejeon, Korea) was used to deposit the chosen metal layers on the glass substrate for the experiment. The deposited metal thickness was measured using an atomic force microscope (AFM) (Veeco Metrology System, Santa Barbara, CA, USA. Model No. 920-006-101).

### 2.2. Preparation of 5-Carboxyfluorescein (FAM)

FAM powder (Sigma-Alrich, ≥95% HPLC, St. Louis, MO, USA) was dissolved directly in ultrapure water (Biosang Inc., Seongnam, Korea). An ultrasonicator (Saehan, SH-2300, Paju, Korea) was used to mix the solutions and accelerate the dissolution of a solid/powder into liquid. A 2 mM solution of FAM was soluble in water and its excitation spectrum peaked at 492 nm (λ_ex_ = 492 nm), while the fluorescence emission spectrum peaked at 518 nm (λ_em_ = 518 nm). The fluorescence experiment was designed to use seven different concentrations (C_FAM_ = 5, 10, 15, 20, 25, 30, and 35 nM), all diluted from 2 mM.

## 3. Results and Discussion

### 3.1. Fluorescence Enhancement for Plasmonic Chips of Different Metal Layers

**Figure 2:**
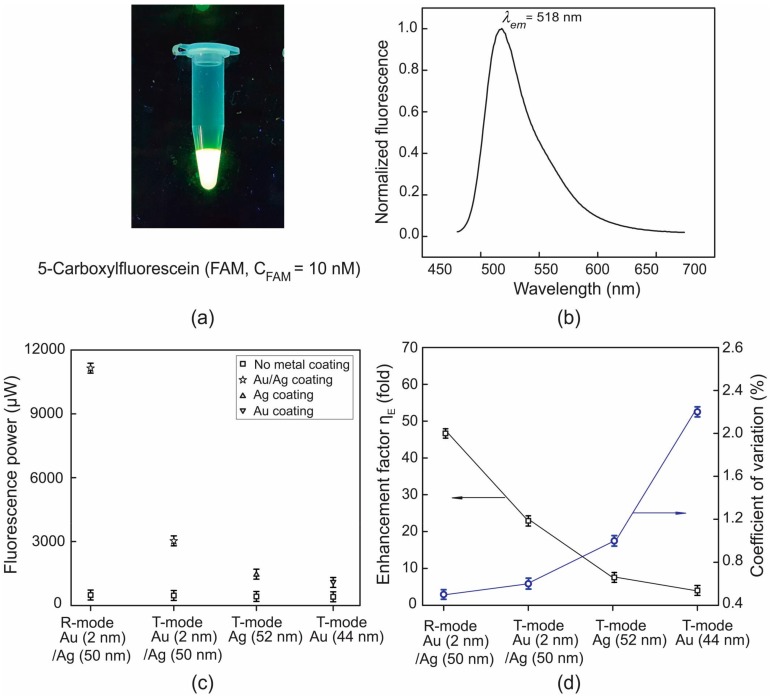
(**a**) A fluorescence image of a 5-carboxyfluorescein (FAM) sample (C_FAM_ = 10 nM); (**b**) an emission spectrum of FAM; (**c**) fluorescence power for various combination of plasmonic chips and setups; (**d**) estimated enhancement factor and the corresponding coefficient of variation (CV).

A conventional fluorescence system enables us to check the presence of the green color of fluorescence, emitting from a FAM sample of concentration C_FAM_ = 10 nM, under the illumination of ultraviolet (UV) light excitation, and its spectrum centers at 518 nm, as shown in Figure 2a,b.

The $\eta_{E}$ estimation was based on a comparison of fluorescence power between the two cases, i.e., the fluorescence obtained with a chip of metal coating and with that of no metal coating. We measured the background power by measuring the optical power with a dye-free deionized water (DI water) filled in a chamber, for a baseline of optical power. From this, the real fluorescence contribution would add up to the optical power to be measured in the case of dye containing chamber [36]. The background power was comprised of autofluorescence from the illuminated region of both a prism, or a slide glass, and the DI water, while detector dark noise was seen to be negligible.

We used four different combinations of plasmonic chips and setups for fluorescence enhancement, as shown in Figure 2c. It was seen that the monometallic film of Ag outdid that of Au for fluorescence enhancement with the T-mode setup. This is due to the fact that, the smaller magnitude of the imaginary part of the relative permittivity of Ag (as compared to that of Au), drives the smaller damping effects of surface plasmons during their propagation, with the consequence of a higher quality factor of the plasmonic resonance. This leads to a higher enhancement of the plasmonic field strength in those regions of the metal surface where the dye molecules are placed. Moreover, on the bimetal plasmonic chip, where a 2 nm-thick Au layer was overlaid on 50 nm-thick Ag layer, to prevent the Ag from direct contact with the liquid buffer, produced higher enhancement than the monometallic Ag layer. It was also found that the bimetal surface plasmons produced a higher field strength than the monometallic Ag layer at λ_em_ (= 518 nm), which was not the case for a longer visible wavelength, e.g., 633 nm.

We also found that the bimetal plasmonic chip could enhance the fluorescence more significantly in the R-mode setup ($\eta_{E}$ = 46.5-fold) than in the T-mode one ($\eta_{E}$ = 22.3-fold), as shown in Figure 2d. This was attributed to the fact that the R-mode setup utilizes plasmonic enhancement at the excitation channels, i.e., at λ_ex_ as well as at λ_em_. Enhanced fields of surface plasmon evanescent light increased the rate of excitation of dye molecules placed within 200 nm above the metal surface.

We also examined the reproducibility of the enhanced fluorescence by repeating the measurement over five duplicate plasmonic chips of each kind, as shown in Figure 2d. Interestingly, the use of Ag as a primary thickness metal in the plasmonic metal layer enhanced its reproducibility (suppressed CV) over the monometallic Au layer. Moreover, the additional overlayer of Au on top of Ag, as in the case of bimetal chips, suppressed the CV, down to 0.5% (markedly high reproducibility), particularly in the R-mode setup. It was obvious that the CV tended to take up a behavior inversely related to that of $\eta_{E}$. This indicated that a higher enhancement of fluorescence was more likely to be reproducible. This may stem from the fact that larger local plasmonic fields were more likely to dominate plasmonic-coupling of dye molecules above a metal surface, thus suppressing the more effectively random nature of surface roughness induced change, in a plasmonic local field.

### 3.2. FAM Concentration-Dependent Enhancement of Fluorescence

**Figure 3:**
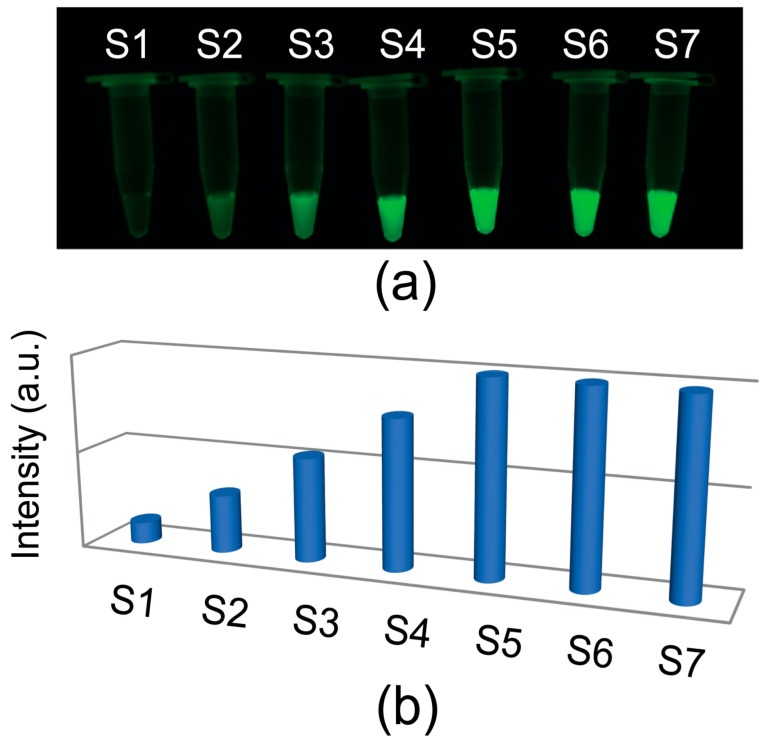
(**a**) A photo for fluorescence image under UV light excitation for various FAM concentrations; (**b**) relative intensities of fluorescence from the corresponding FAM concentrations (S1–FS7 correspond to FAM concentrations, i.e., 5, 10, 15, 20, 25, 30, and 35 nM, respectively).

Figure 3a,b show the image of the C_FAM_-dependent fluorescence and the corresponding intensity obtained in a conventional fluorescence system. Fluorescence signal was detected and analyzed using the Gel Doc EZ system (Bio-Rad, Hercules, CA, USA) and the Image Lab 4.0 software (Bio-Rad). It was clear that fluorescence intensity saturated at C_FAM_ beyond 20 nM, due to photo-bleaching.

**Figure 4:**
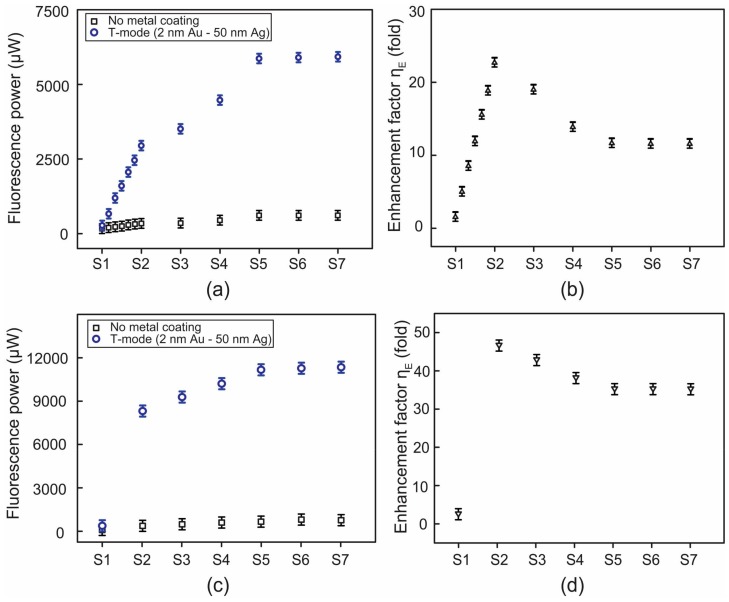
FAM concentrations (C_FAM_)-dependent fluorescence power and fluorescence enhancement factor ($\eta_{E}$). (**a**,**b**) refer to a T-mode setup while (**c**,**d**) to an R-mode one. S1–S7 (see Figure 3) correspond to FAM concentrations, i.e., 5, 10, 15, 20, 25, 30, and 35 nM, respectively.

However, this photo-bleaching disappeared when using a surface plasmon-FAM molecule coupling as shown in Figure 4a–d. It was visible that the use of C_FAM_ beyond 5 nM still amplified fluorescence, by more than 10-fold in both mode setups. We also obtained the linear relation of $\eta_{E}$ versus C_FAM_ between 5 nM and 10 nM (T-mode), as shown in Figure 4a,b. The maximum $\eta_{E}$ of 46.5-fold was achieved at 10 nM (as presented above) in the R-mode. Beyond 10 nM, $\eta_{E}$ decreased and saturated in both T- and R-mode setups, in a similar fashion. In other words, plasmon-FAM molecules coupling amplified fluorescence at all C_FAMs_ used but $\eta_{E}$ did not increase monotonically with the C_FAM_, which is in contrast to Reference [36]. Interestingly, enhanced fluorescence powers, as shown in Figure 4a,c, still exhibited C_FAM_-dependence similar to that of photo-bleached fluorescence, shown in Figure 3b. This might be attributed to the presence of partial FAM molecules, under photo-bleaching, even in the cases of the plasmonic enhancements. This is not the case in the DNA conjugated SYBR Green I [36], but it may be a characteristic feature of FAM molecules. Thus, it implies that a part of FAM molecules undergoes photo-bleaching while the others are responsible for such fluorescence enhancements. This can result in a nonlinear $\eta_{E}$ (that does not increase with C_FAM_) as a sign of the presence of partial FAM molecules subjected to photo-bleaching.

## 4. Conclusions

We demonstrated the enhancement of fluorescence from a fluorescent dye (i.e., FAM), via its coupling with surface plasmons, using two kinds of structures of optical setups, i.e., the T- and R-mode setup. The R-mode setup provided a higher enhancement of fluorescence than the T-mode one due to a plasmonic enhancement of transition rates for both the excitation and the emission channels. We found that the bimetal plasmon coupling produced the maximum $\eta_{E}$ of 46.5-fold with an excellent reproducibility (CV of 0.5%), in the R-mode setup. It was also unveiled that higher plasmonic enhancement of fluorescence was more highly reproducible. We also investigated C_FAM_-dependent fluorescence enhancement in the setups of both modes. Unlike a conventional fluorescence system, no photo-bleaching was observed at all C_FAMs_ used (0 to 35 nM) but $\eta_{E}$ varied nonlinearly with C_FAM_. This nonlinearity may reflect the fact that part of the FAM molecules undergoes photo-bleaching while the others are responsible for fluorescence enhancement through a plasmonic-coupling.

## Supplementary Materials

The following are available online at http://www.mdpi.com/2072-666X/9/9/460/s1, Figure S1: (a) and (b) represent reflectance curves as a function of incident angle for single layer of Au and Ag of various thicknesses, respectively. (c) and (d) represent enhancement of electric field intensity versus distance from the metallic surface in a liquid region at excitation wavelength (λ_ex_ = 470 nm).
