# Supplementary material for: Fluorescence Enhancement Using Bimetal Surface Plasmon-Coupled Emission from 5-Carboxyfluorescein (FAM)
Source: Micromachines (Basel). 2018 Sep 12;9(9):460. doi: 10.3390/mi9090460 (PMC6187710; doi:10.3390/mi9090460)
Supplement: Supplementary file 1 [file micromachines-09-00460-s001.pdf]

Supplementary Materials: Fluorescence Enhancement Using Bimetal Surface Plasmon-Coupled Emission from 5-Carboxyfluorescein (FAM)

Nhu Hoa Thi Tran [1,2], Kieu The Loan Trinh [3], Jun-Ho Lee [4], Won Jung Yoon [5] and Heongkyu Ju [1,2,6,*]

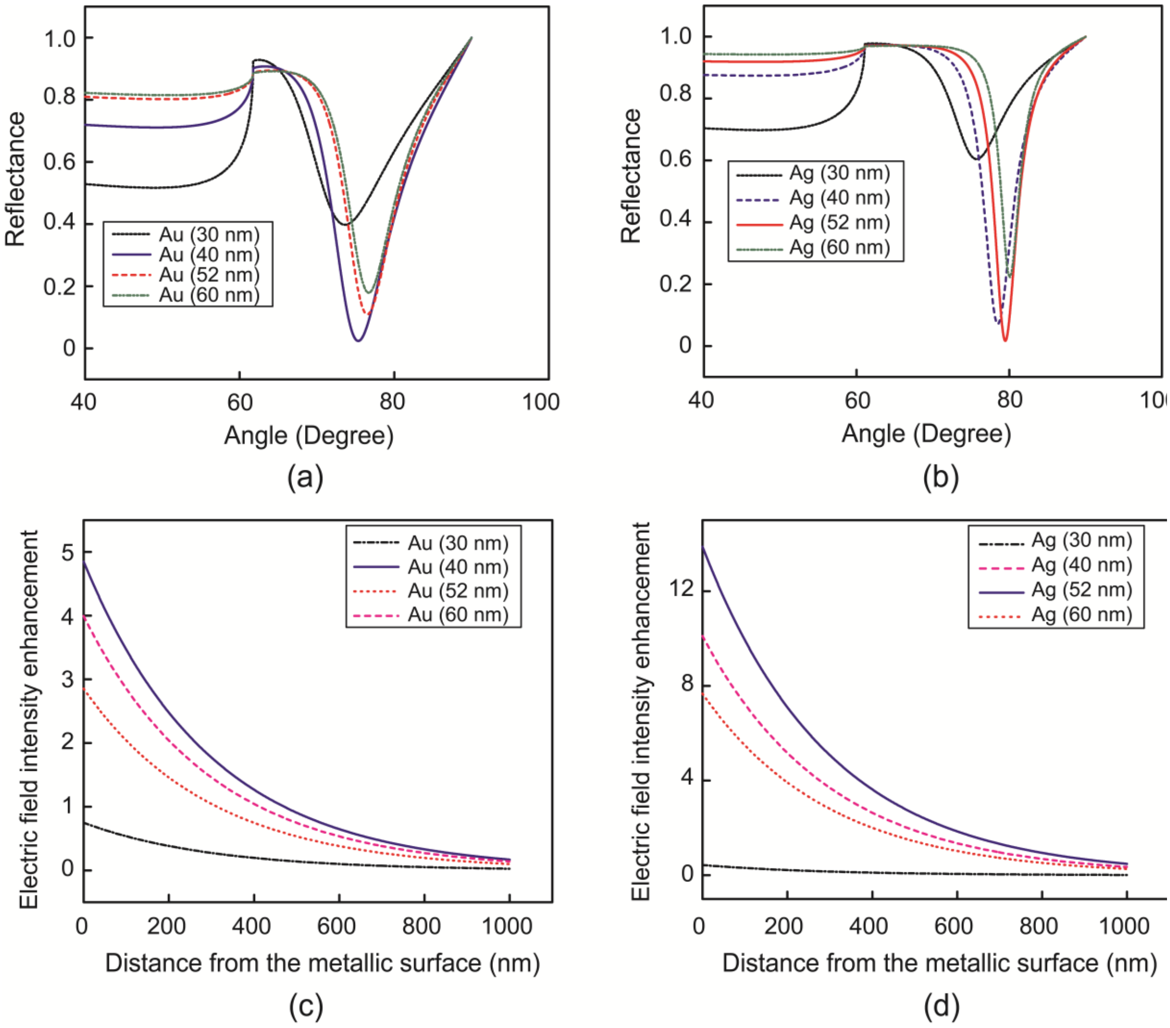

Figure S1. (**a**) and (**b**) represent reflectance curves as a function of incident angle, for a single layer of Au and Ag of various thicknesses, respectively. (**c**) and (**d**) represent enhancement of the electric field intensity versus distance from the metallic surface in a liquid region at the excitation wavelength ($\lambda_{ex} = 470$ nm).

Calculation of Electric Field Intensity Enhancement

We computed the reflectance of light in a Kretschmann-Raether surface plasmon resonance (SPR) configuration, using an optical transfer matrix formula, for a multilayer stack. For a multilayer stack of N layers, labeled as 0 (prism) to N (liquid with fluorophores), we assumed the transverse magnetic polarization of incident light and thus obtained the tangential components of the electric (E_a) and the magnetic (B_a) fields at the boundary between the layers N−1 and N, as follows:

$$\begin{bmatrix} E_a \\ B_a \end{bmatrix} = \prod_{i=1}^{N} \begin{bmatrix} \cos\delta_i & \dfrac{i\sin\delta_i}{\gamma_i} \\ i\gamma_i \sin\delta_i & \cos\delta_i \end{bmatrix} \begin{bmatrix} E_N \\ B_N \end{bmatrix} \tag{1}$$

Here,

$$\delta_i = \left(\frac{2\pi}{\lambda}\right) n_i d_i \cos\theta_i \tag{2}$$

$$\gamma_i = \frac{n_i\sqrt{\varepsilon_0\mu_0}}{\cos\theta_i} \tag{3}$$

Here δ_i is the optical phase introduced by a single traversal of the field across the ith layer. We denoted n_i and d_i as the refractive index and the thickness of the ith layer, respectively. θ_i is the incident angle of light to the ith layer. ε_0 and μ_0 are the permittivity and permeability of free space, respectively. Using $\sum_{i=1}^{N} M_i = m$, where M_i is the individual transfer matrix, the reflection coefficient r for transverse magnetic (TM)-polarized light is then expressed as:

$$r = \frac{\gamma_N m_{11} + \gamma_0\gamma_N m_{12} - m_{21} - \gamma_0 m_{22}}{\gamma_N m_{11} + \gamma_0\gamma_N m_{12} + m_{21} + \gamma_0 m_{22}} \tag{4}$$

$$t = \frac{2\gamma_0(\frac{n_N}{n_0})}{\gamma_N m_{11} + \gamma_0\gamma_N m_{12} + m_{21} + \gamma_0 m_{22}} \tag{5}$$

Then, the field enhancement is given by $\frac{I_2}{I_1} = \sqrt{\frac{\varepsilon_{r2}}{\varepsilon_{r0}}}|t|^2$.
